# Supplementary material for: A proof-of-concept study on the genomic evolution of Sars-Cov-2 in molnupiravir-treated, paxlovid-treated and drug-naïve patients
Source: Commun Biol. 2022 Dec 15;5:1376. doi: 10.1038/s42003-022-04322-8 (PMC9753865; doi:10.1038/s42003-022-04322-8)
Supplement: Supplementary file 2 — Supplementary Information [file 42003_2022_4322_MOESM2_ESM.pdf]

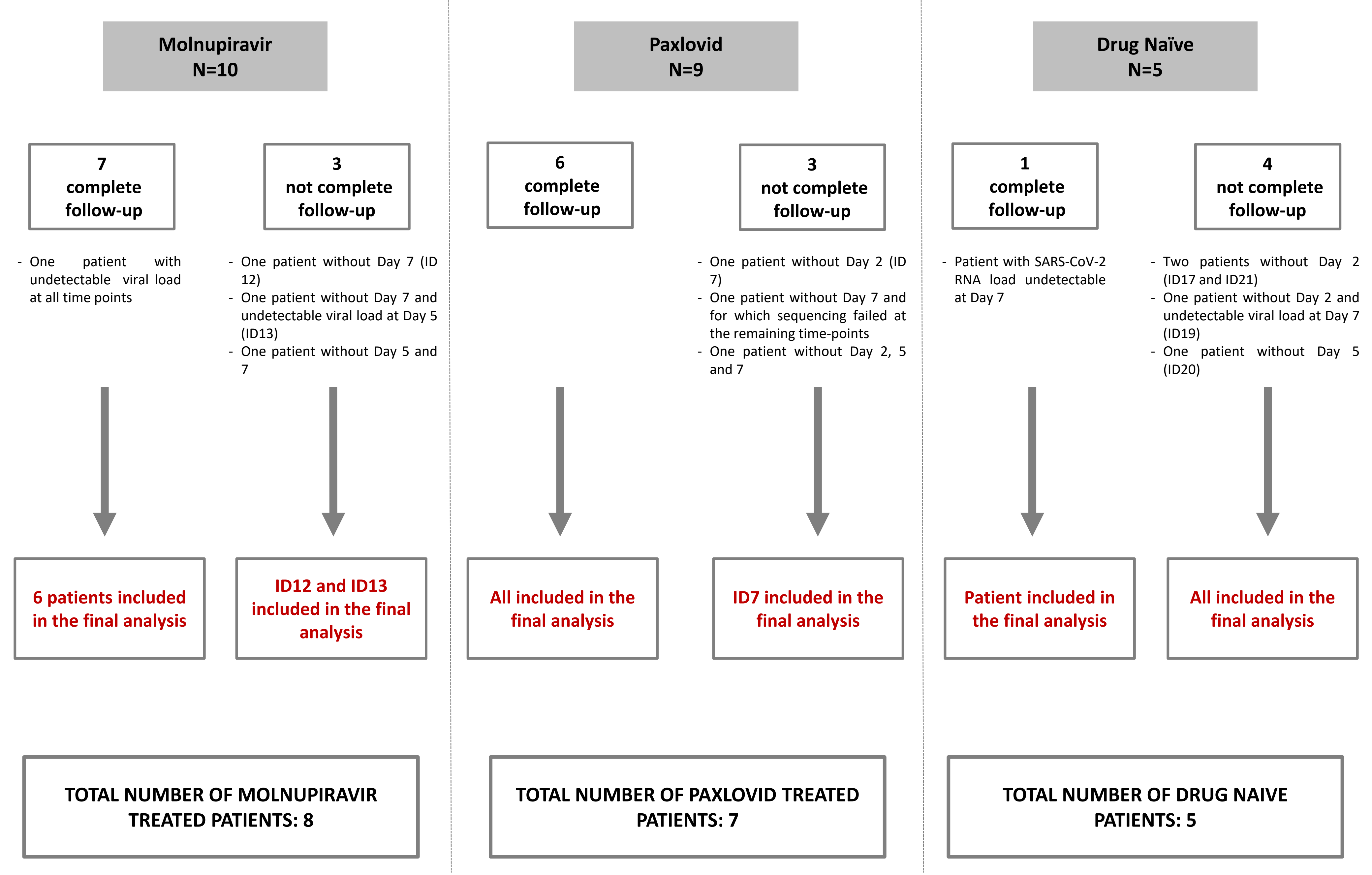

Supplementary Figure 1. Selection criteria for the 20 SARS-CoV-2 infected patients included in the final analyses.

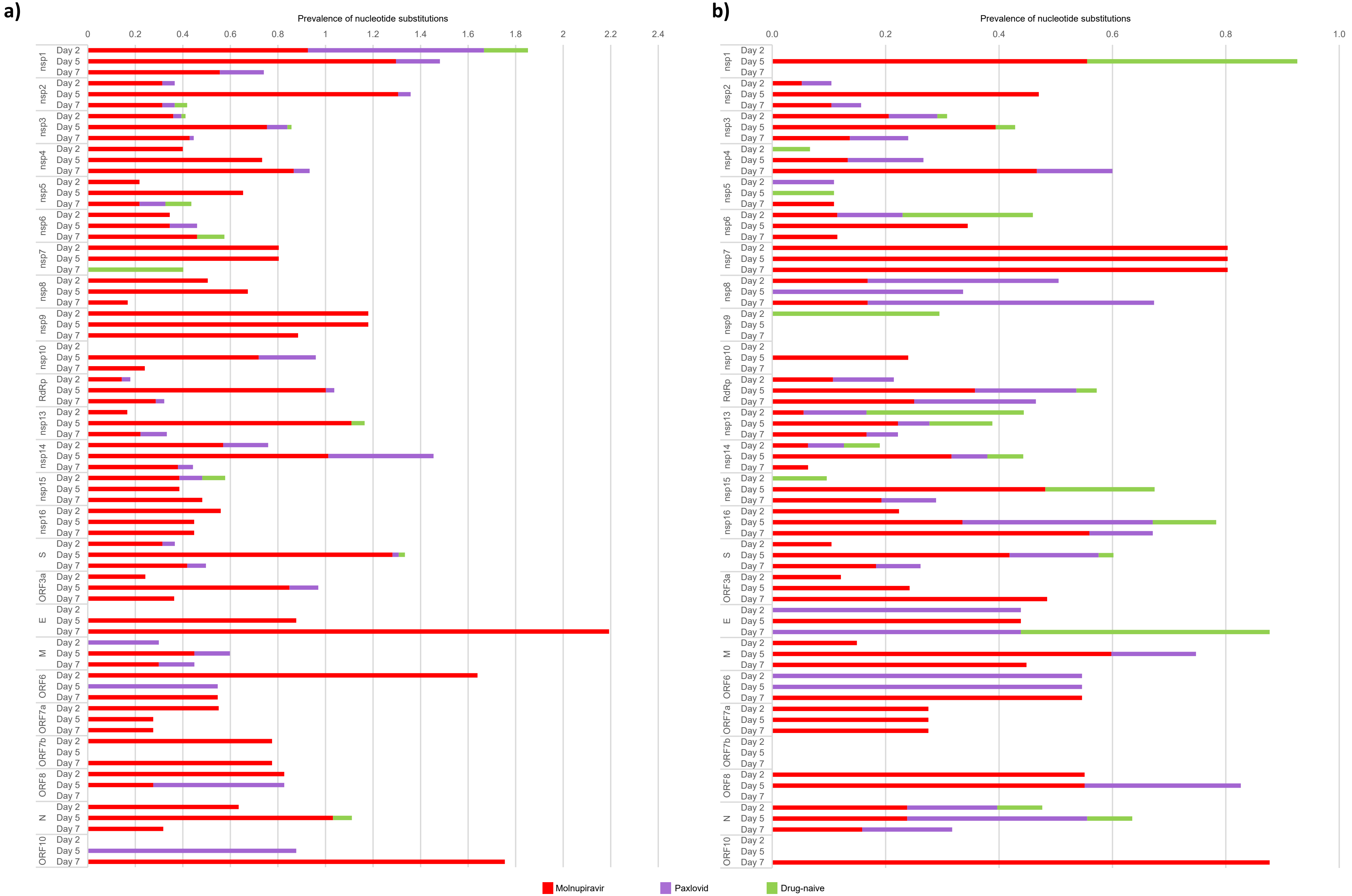

Supplementary Figure 2. Prevalence of C to T and G to A transitions (a) and other nucleotide substitutions (b) per site divided by nucleotide sequence length. Graph bars are colored based on study group.

Supplementary Table 1: Genetic distance between different time-points according to study groups.

	Genetic distance, median (IQR) ($x10^{-4}$)			P-value*	P-value**
	Day 0 - Day 2	Day 2 - Day 5	Day 5 - Day 7		
**Molnupiravir treated**	8.5 (0.0-9.6)	16.2 (8.5-32.9)	20.9 (8.5-29.2)	0.043	0.144
**Paxlovid treated**	2.1 (0.0-3.4)	3.1 (0.9-5.5)	3.1 (0.4-9.0)	0.686	0.600
**Drug Naive**	3. (0.8-6.0)	3.4	2.1 (0.4-3.4)	NA	0.155

Genetic distance is expressed as nucleotide substitutions per site. P-values were calculated by Wilcoxon test; NA: not available. *P-values comparing genetic distance observed at Day0-Day2 and at Day2-Day5. **P-values comparing genetic distance observed at Day2-Day5 and at Day5-Day7.
