# Supplementary material for: A proof-of-concept study on the genomic evolution of Sars-Cov-2 in molnupiravir-treated, paxlovid-treated and drug-naïve patients
Source: Commun Biol. 2022 Dec 15;5:1376. doi: 10.1038/s42003-022-04322-8 (PMC9753865; doi:10.1038/s42003-022-04322-8)
Supplement: Supplementary file 3 — Description of Additional Supplementary Files [file 42003_2022_4322_MOESM3_ESM.pdf]

Description of Additional Supplementary Files

File name: Supplementary Data 1
Description: The source data behind graph of Figure 1.

File name: Supplementary Data 2
Description: The source data behind graphs of Figure 3, 4 and S2.
