# Supplementary material for: A proof-of-concept study on the genomic evolution of Sars-Cov-2 in molnupiravir-treated, paxlovid-treated and drug-naïve patients
Source: Commun Biol. 2022 Dec 15;5:1376. doi: 10.1038/s42003-022-04322-8 (PMC9753865; doi:10.1038/s42003-022-04322-8)
Supplement: Supplementary file 6 — Reporting Summary [file 42003_2022_4322_MOESM6_ESM.pdf]

nature portfolio

Corresponding author(s): Carlo Federico Perno

Last updated by author(s): Nov 18, 2022

Reporting Summary

Nature Portfolio wishes to improve the reproducibility of the work that we publish. This form provides structure for consistency and transparency in reporting. For further information on Nature Portfolio policies, see our Editorial Policies and the Editorial Policy Checklist.

Statistics

For all statistical analyses, confirm that the following items are present in the figure legend, table legend, main text, or Methods section.

n/a	Confirmed	
☐	☒	The exact sample size (*n*) for each experimental group/condition, given as a discrete number and unit of measurement
☐	☒	A statement on whether measurements were taken from distinct samples or whether the same sample was measured repeatedly
☐	☒	The statistical test(s) used AND whether they are one- or two-sided<br>*Only common tests should be described solely by name; describe more complex techniques in the Methods section.*
☒	☐	A description of all covariates tested
☒	☐	A description of any assumptions or corrections, such as tests of normality and adjustment for multiple comparisons
☐	☒	A full description of the statistical parameters including central tendency (e.g. means) or other basic estimates (e.g. regression coefficient) AND variation (e.g. standard deviation) or associated estimates of uncertainty (e.g. confidence intervals)
☐	☒	For null hypothesis testing, the test statistic (e.g. *F*, *t*, *r*) with confidence intervals, effect sizes, degrees of freedom and *P* value noted<br>*Give P values as exact values whenever suitable.*
☒	☐	For Bayesian analysis, information on the choice of priors and Markov chain Monte Carlo settings
☒	☐	For hierarchical and complex designs, identification of the appropriate level for tests and full reporting of outcomes
☒	☐	Estimates of effect sizes (e.g. Cohen's *d*, Pearson's *r*), indicating how they were calculated

Our web collection on statistics for biologists contains articles on many of the points above.

Software and code

Policy information about availability of computer code

Data collection	No software for data collection was used
Data analysis	Python v3.7<br>Fastp (v0.20.1) for fastq triiming<br>BWA-mem for sequence alignment<br>samtools/bcftools for variant calling<br>vcf_consensus_builder for consensus generation<br>Pangolin (v4.1.1) for SARS-CoV-2 variants assignement<br>MAFFT (v7.475) for multiple sequence alignment<br>Bioedit V7.2<br>MEGA (v11) for genetic distance and dS/dN calculations<br>IqTree2 (v2.1.3) for maximum likelihood phylogeny tree construction<br>iTOL for tree annotation<br>Circos package (v0.69-9) for circus plots construction<br>SPSS Software (23.0) for statistical analysis<br>GraphPad Prism 8

For manuscripts utilizing custom algorithms or software that are central to the research but not yet described in published literature, software must be made available to editors and reviewers. We strongly encourage code deposition in a community repository (e.g. GitHub). See the Nature Portfolio guidelines for submitting code & software for further information.

Data

Policy information about availability of data

All manuscripts must include a data availability statement. This statement should provide the following information, where applicable:

- Accession codes, unique identifiers, or web links for publicly available datasets
- A description of any restrictions on data availability
- For clinical datasets or third party data, please ensure that the statement adheres to our policy

Nature Research reporting summary was uploaded as related manuscript file. The source data behind Figure 1 and Figure 3, 4 and Supplementary Figure 2 were provided as Supplementary Data 1 and Supplementary Data 2, respectively. The SARS-CoV-2 sequence data obtained for this study are openly available on European Nucleotide Archive (ENA) under the accession numbers ERR10442739, ERR10442746-47, ERR10442752-2801, ERR10442804, ERR10442809-10, ERR10442815-18, ERR10442825-28, and ERR10442833 (Project ERP142142). The data regarding demographic and clinical features related to each patient are available on request from the corresponding author. The data are not publicly available because they may contain information that could compromise privacy.

Human research participants

Policy information about studies involving human research participants and Sex and Gender in Research.

Reporting on sex and gender	Only information regarding sex is reported in the manuscript. The data regarding sex and other demographics and clinical features related to each patient are available on request from the corresponding author. The data are not publicly available because they may contain information that could compromise privacy.
Population characteristics	From March 2022 to May 2022, nasopharyngeal swabs at four different time points (Day 0, Day 2, Day 5 and Day 7) were available from 19 adult subjects treated with either Molnupiravir or Paxlovid at the Azienda Ospedaliero-Universitaria Policlinico of Modena, and from 5 drug-naïve paediatric subjects at the Bambino Gesù Children Hospital IRCCS in Rome. Eleven individuals (55.0%) were male, and 18 (90.0%) had at least one comorbidity. For both Molnupiravir and Paxlovid, treatment was started at Day 0 and ended at Day 5. Complete follow-up or SARS-CoV-2 whole genome sequencing at each time point was available for 20 individuals (8 for Molnupiravir, 7 for Paxlovid and 5 for drug-naïve).<br>Whole genome sequencing analysis revealed that all SARS-CoV-2 infections belonged to Omicron Clade, BA.2 sublineage. Half of them (N=10, 50%) belonged to lineage BA.2.9, followed by pure BA.2 (N=4, 20%), BA.2.12/BA.2.12.1 (N=3, 15%) and BA.2.3/BA.2.3.15 (N=3, 15%). Median (Interquartile range, IQR) date of symptoms' start, and first positivity were 2 May 2022 (27 April 2022 - 12 May 202) and 02 May 2022 (28 Apr 2022 - 11 May 2022), respectively.<br>Among differences among the 3 study groups, age and prevalence of comorbidities were higher in Molnupiravir and Paxlovid treated individuals respect to drug-naïve (P=0.005 and 0.032). Molnupiravir-treated and Paxlovid-treated patients had a first positivity date more recent respect to drug-naïve individuals (P=0.033), and the prevalence of the sublineage BA.2.9 was more prevalent in Molnupiravir-treated and drug-naïve individuals compared to Paxlovid-treated patients (P=0.002). Patients characteristics were described in the Manuscript, Result section, Population characteristics paragraph and Table 1.
Recruitment	This retrospective observational proof-of-concept study initially included 24 individuals diagnosed for SARS-CoV-2 infection at Azienda Ospedaliero-Universitaria Policlinico of Modena and Bambino Gesù Children Hospital IRCCS from March 2022 to May 2022. Of these, 10 individuals started SARS-CoV-2 antiviral treatment with Molnupiravir, 9 with Paxlovid, and 5 individuals received no treatment (drug-naïve), depending on physicians' discretion.<br>For each individual with nasopharyngeal swabs available, SARS-CoV-2 viral load and genomic variability were retrospectively analyzed at 4 time points: before SARS-CoV-2 antiviral treatment start (Day 0), 2 days after SARS-CoV-2 antiviral treatment start (Day 2), 5 days after SARS-CoV-2 antiviral treatment start, i.e. end of treatment (Day 5), and 2 days after treatment end (Day 7). The same data were obtained at 4 comparable time points for the 5 drug-naive individuals.<br>Based on availability of samples at each time point, results on SARS-CoV-2 viral load quantification and whole-genome SARS-CoV-2 sequencing, 20 individuals were finally selected for the study, and divided in three study groups according to treatment as follows: 8 Molnupiravir-treated, 7 Paxlovid-treated and 5 drug-naïve individuals. Recrutiment strategy was described in the Manuscript, Method section, Study population paragraph and Supplementary Figure 1.
Ethics oversight	The study protocol was approved by local Research Ethics Committee of the Azienda Ospedaliero-Universitaria Policlinico of Modena and Bambino Gesù Children hospitals (prot. 396/2020/OSS/AOUMO and prot. 2384_OPBG_2021). This study was conducted in accordance with the principles of the 1964 Declaration of Helsinki. Informed consent was waived in accordance with the hospital regulations on retrospective observational studies.

Note that full information on the approval of the study protocol must also be provided in the manuscript.

Field-specific reporting

Please select the one below that is the best fit for your research. If you are not sure, read the appropriate sections before making your selection.

☒ Life sciences ☐ Behavioural & social sciences ☐ Ecological, evolutionary & environmental sciences

For a reference copy of the document with all sections, see nature.com/documents/nr-reporting-summary-flat.pdf

Life sciences study design

All studies must disclose on these points even when the disclosure is negative.

Sample size	Due to the proof-of-concept nature of this study no sample size calculation was performed.
Data exclusions	The selection criteria for the 20 SARS-CoV-2 infected patients included in the final analyses are reported in Supplementary Figure 1. Four patients were excluded because swabs available at few time points (n=3), or undetectable viral load at all time points (n=1).
Replication	The generation of whole genome sequences was not repeated. All the SARS-CoV-2 sequences finally considered for this study were obtained by previously validated method (https://doi.org/10.1002/jmv.27559), and are of excellent quality in term of depth and qscore (average q28 threshold and read length > 129 nt). Therefore they are fully reliable.
Randomization	Randomization is not relevant for this study. Treatment decision depends on physicians' discretion.
Blinding	Data analysis was performed in blind.

Reporting for specific materials, systems and methods

We require information from authors about some types of materials, experimental systems and methods used in many studies. Here, indicate whether each material, system or method listed is relevant to your study. If you are not sure if a list item applies to your research, read the appropriate section before selecting a response.

Materials & experimental systems

n/a	Involved in the study	
☒	☐	Antibodies
☒	☐	Eukaryotic cell lines
☒	☐	Palaeontology and archaeology
☒	☐	Animals and other organisms
☒	☐	Clinical data
☒	☐	Dual use research of concern

Methods

n/a	Involved in the study	
☒	☐	ChIP-seq
☒	☐	Flow cytometry
☒	☐	MRI-based neuroimaging
